# Respiratory Abnormalities in Parkinson’s Disease: What Do We Know from Studies in Humans and Animal Models?

**DOI:** 10.3390/ijms23073499

**Published:** 2022-03-23

**Authors:** Katarzyna Kaczyńska, Magdalena Ewa Orłowska, Kryspin Andrzejewski

**Affiliations:** Department of Respiration Physiology, Mossakowski Medical Research Institute, Polish Academy of Sciences, Pawińskiego 5 St., 02-106 Warsaw, Poland; morlowska@imdik.pan.pl

**Keywords:** Parkinson’s disease, respiratory dysfunction, apnea, dyspnea, hypercapnia, hypoxia

## Abstract

Parkinson’s disease (PD) is the second most common progressive neurodegenerative disease characterized by movement disorders due to the progressive loss of dopaminergic neurons in the ventrolateral region of the substantia nigra pars compacta (SNpc). Apart from the cardinal motor symptoms such as rigidity and bradykinesia, non-motor symptoms including those associated with respiratory dysfunction are of increasing interest. Not only can they impair the patients’ quality of life but they also can cause aspiration pneumonia, which is the leading cause of death among PD patients. This narrative review attempts to summarize the existing literature on respiratory impairments reported in human studies, as well as what is newly known from studies in animal models of the disease. Discussed are not only respiratory muscle dysfunction, apnea, and dyspnea, but also altered central respiratory control, responses to hypercapnia and hypoxia, and how they are affected by the pharmacological treatment of PD.

## 1. Introduction

**Figure 1:**
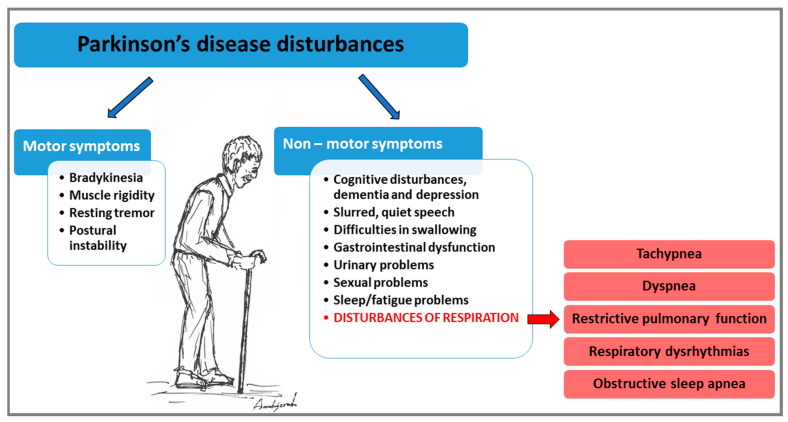
Motor and non-motor symptoms appearing in Parkinson’s disease (PD). Respiratory impairments are present among the non-motor symptoms.

Idiopathic Parkinson’s disease (PD) is the second most common progressive neurodegenerative disorder caused by the degeneration of dopaminergic (DA) neurons in the midbrain, affecting older adults [1,2,3]. Progressing loss of DA neurons in the ventrolateral area of substantia nigra pars compacta (SNpc) and α-synuclein protein accumulation (known as Lewy bodies) in the remaining neurons lead to reduced ease of voluntary movements as PD develops [4,5,6]. The manifestations of PD consist in a gradual loss of well-defined motor functions and underappreciated non-motor functions that affects individuals to varying degrees [7]. The cardinal motor features of PD are the appearance of resting and postural tremor, muscular rigidity, muscle weakness, bradykinesia, and postural instability [1,7,8]. Non-motor symptoms such as mood changes, depression, anxiety, cognitive decline, hyposmia, constipation, pain, sleep disturbances, and autonomic dysfunction often accompany motor deficits in PD (Figure 1) [6,9,10,11]. They are correlated with the severity of the disorder, but some of them such as depression, olfactory disturbances, and fatigue can occur early in the premotor phase [6,12].

In addition to the previously mentioned impairments, patients with PD exhibit voice and speech abnormalities such as breathy phonation, hoarseness, imprecise articulation, reduced loudness and prosody, dysfunctions that are directly related to upper airway breathing disorders [13,14,15,16]. The breathing problems that occur in PD not solely are related to upper airway muscle dysfunction they also may be associated with changes in other respiratory muscles and the central nervous system (CNS) [17,18,19].

Respiratory disturbances occurring in PD have recently attracted more interest, but their mechanisms are still not understood [20].

In this review, we made an attempt to summarize the available data having regard for the most important and recent ones. We focused on similar observations; however, major discrepancies are also pointed out. Animal studies contributing to the growing understanding of the mechanisms involved in respiratory impairments in PD are also taken into consideration.

## 2. Respiratory Dysfunction in PD

The first clinical description of respiratory distress—“He fetched his breath rather hard”—was given by James Parkinson in 1817 in his An Essay on the Shaking Palsy [8]. Two hundred years since Parkinson’s publication, numerous and varied signs and symptoms of pulmonary dysfunction have been described depending on the portion of the respiratory system affected and the severity of the disease. They have been described as a combination of dysrhythmic breathing pattern, tachypnea, dyspnea, decreased respiratory pressure, sleep disordered breathing, and reduced exercise tolerance (Figure 1) [17,21,22,23,24,25,26,27]. In general, the respiratory disturbances seen in individuals with PD differ widely, from lack of impairments to stridor or severe dyspnea [28]. One of the problems in interpreting the data derives from the discrepancies in the methodologies used, as studies are conducted on heterogeneous groups and not always take into account the effect of antiparkinsonian drugs or stage of the disease [25,28]. Impaired breathing is primarily associated with postural changes, respiratory muscle weakness, and changes in upper airway muscle activation and coordination [24,29,30,31]. However, debate continues as to whether the muscular component is the primary factor responsible for respiratory dysfunction. Certainly, the leading cause of death among PD patients is aspiration pneumonia due to swallowing dysfunction and impaired cough reflex [32,33,34,35,36].

Identifying respiratory disturbances associated with increased morbidity and mortality in PD is important for understanding how pulmonary complications occur and may contribute to the search for new therapeutic strategies to prolong and improve the patients’ quality of life.

## 3. Pulmonary Function and Respiratory Muscle Strength

### 3.1. Human Studies

The importance of respiratory muscle strength in the pathological features of the respiratory abnormalities in Parkinson’s disease was highlighted by clinical and physiological observation. Respiratory muscle strength is analyzed by measuring the maximum inspiratory pressure (MIP) and the maximum expiratory pressure (MEP) [37,38]. Spirometry tests that measure various lung function such as vital capacity (FVC), maximum voluntary ventilation (MVV), and forced expiratory volume in one second (FEV1) can be helpful in assessing respiratory muscle performance. Decreased values of the parameters mentioned describe limitations of the forced respiratory muscle movements in PD that are usually correlated with a poor coordination or rigidity and bradykinesia [37,38,39].

One of the most commonly identified causes of respiratory distress in PD is a dysfunction of the upper airway musculature [39]. In support, significant proportion of patients with parkinsonism presented physiological and symptomatic evidence of upper airway obstruction with limited airflow [29,40,41,42]. According to Sabate et al. [41], bradykinesia appeared more frequently in PD patients with upper airway obstruction than in those without respiratory distress. Moreover, approximately 70% of PD subjects manifest hypophonia, which is the most common manifestation of upper airway obstruction [21,43].

An electromyogram study of the major respiratory muscle exhibited a close-to-normal diaphragm activity in patients with PD, ruling out its involvement in respiratory disturbances [44]. Therefore, the impaired activity of other respiratory muscles, such as inspiratory intercostal muscles, was assumed [45]. Inspiratory muscles weakness, assessed during spirometry, was afterwards confirmed in early-stage [22,31], mild to moderate [23,24,46], and late-stage PD [24,47]. Recent promising reports indicate that respiratory muscle strength in older adults with PD can be improved by regular muscle training, which also confirms that, at least in part, respiratory muscle weakness is responsible for respiratory distress in this disease [48,49,50,51].

Respiratory problems may already manifest at the beginning of the disease [19,31] or remain unnoticed because of the prevalence of motor disability [45,52]. Nevertheless, bradykinesia and rigidity, two major features of PD that worsen with disease progression, appear to correlate with the deterioration of pulmonary function and respiratory muscle strength [24,31,37,38]. Presumably, they lead to a gradual decrease in chest wall compliance due to rib cage stiffness and reduced range of motion, but also to decreased muscle elasticity, and consequently reduced tidal volume [23,37,38,53,54]. Kaminsky et al. [55] have shown that lung function in PD patients deteriorates after one year compared to healthy controls. The parameters that declined were FEV1 and FVC, with little change in FEV1/FVC, indicating a reduction in lung volume associated with respiratory muscle weakness rather than the development of airway obstruction.

Abnormalities of the respiratory function of both types, obstructive and restrictive, were reviewed and analyzed in PD patients previously [26,56]. Both reviews also summarized in detail lung function studies in PD, showing the frequency of these patterns. The obstructive type occurred in less than 50% of the cases studied [41,53,54]. In contrast, the restrictive type seemed to predominate as in spirometry studies, it was observed in more than 60% [57] and even 80% of PD subjects [37,39,58]. The reason of the prevalence of the restrictive respiratory pattern in PD patients may be related not only to increased chest wall rigidity and impaired respiratory muscle activity but also to diminished lung volume due to kyphoscoliosis (excessive lateral and posterior curvature of the spine) common in PD patients and also to abnormal respiratory control [41,56,59,60]. All these factors can lead to chest wall asynchrony and inspiratory paradoxical breathing movement, which have been demonstrated in half of the patients studied in one case [59]. In addition, the restrictive pattern seems to intensify in the “off” state and in more advanced stages of the disease, due to increased chest wall muscle rigidity and decreased chest mobility [37,61]. The terms “on” and “off” describe different stages of motor fluctuations. The “on” state is when PD patients experience a good motor function. The “off” state is when the symptoms of PD return [62].

### 3.2. Animal Studies

The lung function tested via cineradiography in a mouse hemi-PD model with 6-hydoxydopamine (6-OHDA) striatal injection, showed decreased diaphragm mobility [63]. In a bilateral rat model triggered in a similar way, reduced resting and hypercapnia-induced frequency of diaphragm electromyogram was observed [64]. These studies contradict observations in humans, in which diaphragm function remained normal [45,65,66], demonstrating that animal models may imperfectly reproduce Parkinson’s disease signs.

## 4. Obstructive Sleep Apnea (OSA)

It appears that upper airway dysfunction such as rigidity and bradykinesia in PD patients may promote obstructive sleep apnea (OSA), a disorder characterized by recurrent upper airway obstruction during sleep, leading to periods of cessation of breathing, intermittent hypoxemia, and sleep fragmentation [27,67]. However, divergent opinions exist regarding the incidence of OSA-related events in PD, ranging from a lower incidence [68] and a similar risk of apnea [69,70,71,72] to a higher prevalence than in control subjects [73,74]. Consistent with this latter assertion is a recent systematic review and meta-analysis of more than 60 polysomnographic studies that showed an increased index of apnea/hypopnea in patients with PD [75]. Discrepancies between studies may be related to the variable definition of hypopnea, the effects of medications, the “off” or “on” status, or a biased selection of the control groups. For example, individuals without PD typically have a higher BMI than affected subjects. Recent findings using polysomnography have shown that OSA may affect between 27% and over 60% of patients with PD [76,77,78,79,80]. Unfortunately, most of these studies have some limitations, such as the lack of control groups. Therefore, uncertainty remains as to whether OSA is a feature of PD [30].

Interestingly, some researchers reverse the cause-and-effect relationship and indicate that patients with OSA may have an increased risk of developing PD. Several longitudinal cohort follow-up studies conducted in the Taiwanese population found an association between OSA and PD and indicated that patients with OSA are at a significant risk for developing Parkinson’s disease [81,82,83]. A recent study is consistent with these observations, as the authors observed higher plasma levels of total and phosphorylated α-synuclein in OSA patients and suggested that chronic intermittent hypoxia increases α-synuclein levels, contributing to the pathogenesis of PD [84]. A detailed discussion of the pathophysiology of OSA in PD was included in a recent review paper [27].

## 5. Central Respiratory Control

### 5.1. Human Studies

Despite the respiratory muscle dysfunction mentioned above, a fundamental question still remains: are some of the respiratory problems in PD due to brain deficits in the respiratory drive to the muscles? Zhang et al. [19] showed an increase in P0.1 values in patients with early-stage idiopathic PD, which indicates abnormalities in the respiratory control center. P0.1 measurement of the airway occlusion pressure (the negative airway pressure generated during the first 100 ms of an occluded inspiration) is an indicator of central respiratory drive related to the level of inspiratory muscle activity [85,86]. Conclusion has been drawn that a decrease in respiratory muscle strength expressed by a decrease in maximal inspiratory and expiratory pressures results in compensation and in an increase in central respiratory output to maintain the normal physiological ventilation. [19].

Little effort has been made to investigate abnormalities of the brain respiratory centers of PD patients. In a very rare hereditary form of parkinsonism with central hypoventilation—Perry syndrome—liosis and neuronal loss have been demonstrated in multiple brainstem nuclei [87,88]. Changes explaining the central respiratory dysfunction were found in multiply brainstem nuclei involved in breathing control and regulation, such as the dorsal motor nucleus of the vagus (DMV), the solitary tract nucleus (NTS), the nucleus ambiguous (NA), the nucleus retroambiguous (NRA) [87], as well as in the pre-Bötzinger complex (pre-BötC) and in the medullary raphe [88].

Unfortunately, little information is available on brainstem abnormalities in idiopathic PD patients to explain the cause of the respiratory distress. One of the earliest studies showed neuronal loss of neurokinin-1 receptor-like-immunoreactive (NK1R) neurons in human PD ventrolateral medulla [89]. In particular, pre-BötC neurons, which play a key role in respiratory rhythmogenesis, express NK1R [90,91], so their loss should explain the respiratory dysfunction in PD. Further, deficits of TH-positive neurons (dopaminergic and noradrenergic) were shown in idiopathic subjects in two brainstem nuclei, i.e., the DMV, sending efferent fibers that transmit parasympathetic signals to the heart and lungs [92,93,94], and the locus coeruleus (LC), involved in the central chemoreception [94,95]. Deficits in noradrenaline release in LC are well known to precede the loss of dopamine in the nigrostriatal pathway that occurs in PD [96,97]. Degenerative changes in the LC occur early and are large, so it appears that the loss of noradrenergic neurons, which are chemosensitive and whose axons reach the brainstem, may affect breathing in PD patients.

Another system affected by pathological changes in PD is the serotonergic one, including the median raphe nuclei containing the serotonergic neurons of the caudal brainstem [7,98], which innervate pharyngeal motoneurons and are involved in chemoreception and in maintaining a normal rhythmic respiration [99,100]. However, previous PET studies in humans with PD failed to demonstrate the involvement of the serotonergic system in the development of sleep disorders. PET imaging measuring caudal brainstem serotonin transporter (SERT) displayed significant variations in binding in PD patients but did not predict the severity of sleep disorders and airway dysfunctions [101].

More recent findings using diffusion tensor imaging have shown that alterations of the brainstem and subcortical areas can be observed at the time of PD diagnosis and extend to cortical and white matter areas after one year [102]. Lee et al. [103] demonstrated gray matter volume deficits within the brain autonomic network on MR imaging that correlated with increased airflow resistance and airway obstruction in PD patients. The latest study using high-resolution PET imaging demonstrated synaptic loss not only in the substantia nigra but also in the noradrenergic LC, confirming the presence of neurodegeneration in the brainstem nondopaminergic nucleus [104], which is well known to modulate the ventilatory response to hypercapnia [95].

Another pathological change observed in the brains of PD patients are Lewy bodies–α-synuclein intracytoplasmic aggregates [105], which have been found not only in susceptible neurons of the substantia nigra [106] but also in brainstem structures such as those involved in chemoreception, the raphe nuclei and the LC [107,108,109,110], as well as in the DMV [106,110] and the cholinergic mesopontine tegmentum [110].

The DMV has been displayed to be one of the brainstem nuclei most vulnerable to damage in PD [111,112]. According to Braak’s hypothesis, the development of PD pathology is caudo-rostral and begins at the DMV, where α-synuclein aggregation occurs early in disease progression, even before SNpc [4]. In addition, the vagus nerve fibers interfacing the parasympathetic control of the heart, lungs, and gastrointestinal tract form a close connection between the central and the peripheral nervous systems where PD pathology may begin, according to a recent hypothesis [113]. Interestingly, Lewy-type pathology and axonal degeneration were found in the peripheral sensorimotor branch of the vagus nerve innervating the pharynx. The density of α-synuclein-positive lesions was significantly higher in subjects with documented dysphagia [114,115,116].

### 5.2. Animal Studies

Interesting information about changes in brain centers responsible for the control/regulation of breathing has come from studies in animal models, although these may not necessarily reflect the changes found in idiopathic PD in humans. When it comes to animal models with confirmed degeneration of dopaminergic neurons evoked by lesion with 6-OHDA, altered activity of the hypoglossal (HG) and phrenic (PHR) nerves in the form of magnified amplitudes during hypoxia was reported [116,117]. Changed activity of both nerves innervating the upper airway and the main respiratory muscle may indicate altered central respiratory drive, as postulated in human research [75]. Indeed, studies in the 6-OHDA model, in addition to a significant loss of dopamine in the striatum, described a significant deficit of serotonin in the striatum and brainstem after neurotoxin administration into the medial forebrain bundle (MFB) [118] and small decreases in noradrenaline and serotonin in the striatum but a significant decrease in the noradrenaline level in the brainstem after intracerebroventricular injection of 6-OHDA [117]. Thus, it appears that 6-OHDA treatment may have different effects depending on the administration site on the loss of monoamines in the brainstem, which may affect the respiratory control differently.

Other studies in a rat model of 6-OHDA with injection into the striatum showed a significant reduction in the density of neurokinin-1 receptor-immunoreactive neurons pre-BötC and phox2b-expressing neurons in important brainstem chemosensitive regions such as the NTS and the retrotrapezoid nucleus (RTN) [119,120,121,122]. These changes were accompanied by a decrease in both resting frequency of breathing and tachypneic response to central chemoreflex activation. In a further study using the 6-OHDA model, loss of astrocytes was observed in key regions engaged in respiratory activity such as RTN, NTS, and pre-BötC [120]. Importantly, astrocytes have previously been described as sensors of rapid changes in oxygen levels [123] and as H+ sensors involved in chemoreception through purinergic signaling [124]. All these described abnormalities in brainstem respiratory centers may explain the described changes in the respiratory pattern in the animal model, which, we emphasize, do not necessarily reflect the pathology characteristic of idiopathic PD. For a more detailed discussion of changes in the respiratory centers in a rat model based on 6-OHDA, see the review by Aquino et al. [18].

## 6. Dyspnea

When describing the respiratory problems that occur in Parkinson’s disease, dyspnea cannot be overlooked because it can have a major impact on patients’ quality of life. The incidence of dyspnea, experienced as breathlessness, increases with disease severity and during the off-medication condition [26,125]. It ranges, according to various estimates, from 11% [126] to 40% [127,128], but can easily be underestimated by clinicians who focus mainly on motor disorders. Moreover, the patients themselves do not report this problem because of the very subjective feeling of respiratory discomfort and the difficulty in describing the symptoms of dyspnea. Although the pathological basis of dyspnea in PD is still unclear, it seems that it may be conditioned by a pathology of both central (brainstem ventilatory centers) and peripheral structures (upper airway, respiratory muscles, chest wall, chemoreceptors) involved in the respiratory process [26], as well as by emotional problems of PD patients, such as anxiety and depression [129]. Nonetheless, a nonpharmacological intervention consisting of exercises to strengthen the respiratory muscles has been described to be an effective tool in reducing the sensation of dyspnea [50]. Psychological interventions that include breathing exercises (e.g., mindfulness-based) are also feasible for people with PD and may help control the feelings of shortness of breath caused by anxiety [130].

## 7. Chemical Regulation of Breathing in PD

### 7.1. Normoxic Breathing Pattern

An unchanged normoxic/normocapnic (normal concentration of oxygen in the atmosphere and carbon dioxide in the blood) breathing pattern has been described in PD patients in the early phase of the disease, with a mild condition (Table 1) [131,132,133,134]. Only one study showed a decrease in resting alveolar ventilation, and this difference may be due to the heterogeneity of the study groups and the different stages of the disease [135].

### 7.2. Hypercapnia

CO_2_ is the most important and powerful respiratory stimulus, and central respiratory control is based on the existence of CO_2_/H^+^ ion-sensitive areas located in the brainstem such as the retrotrapezoid nucleus (RTN,) medullary raphe serotonergic neurons, noradrenergic neurons of the LC, and the NTS [95,137,138,139,140,141]. They are responsible for 70–80% of the ventilatory response to hypercapnia [142], the rest of the response being due to carotid body stimulation [143]. A diverse range of ventilatory responses to hypercapnia have been observed in PD patients, ranging from unchanged [132] to reduced [133] and even stimulated [131,136], likely due to the different disease stages in the patients studied and their different experimental conditions (Table 1). An increased ventilatory response to hypoxic hypercapnia was observed in more advanced stage III–IV PD [131] according to the Hoen and Jahr scale and has been linked to DA deficiency in the CNS, indicating an inhibitory effect of DA on the respiratory drive [136]. According to the latter suggestion, patients with stage I–III PD showed an impaired hypercapnic ventilatory response during optimal dopaminergic treatment compared with controls [133]. In another study, Secombe et al. [134] analyzed patients with a mild–moderate stage of PD (I–II). This time, the ventilatory response to hypercapnia did not differ between PD patients and healthy controls, indicating that the earlier the stage of PD, the less the changes in this response.

### 7.3. Hypoxia

The peripheral chemical regulation of breathing depends mainly on carotid bodies (CB), which are sensitive to decreases in arterial partial pressure of oxygen and, to a much lesser extent, to an increase in CO_2_ and arterial blood acidosis [144,145]. CB chemoreceptors transmit visceral information to the caudal portion of the NTS, a key nucleus that relays hypoxic sensory input to other respiratory brain centers [146,147].

The ventilatory response to hypoxia in PD patients is quite discrepant in published studies (Table 1). The two earliest papers described an increased hypoxic ventilatory response (HVR) in PD patients (III–IV stage) compared to controls [131,136]. On the contrary, Seccombe et al., [134] analyzed patients with mild–moderate PD [stage I–II] and found an unchanged HVR. Serebrovskaya et al. [135] studied patients in the more advanced stages of PD [I–III] and showed a decrease in alveolar ventilation during breathing a hypoxic mixture, suggesting abnormal peripheral chemoreception.

Further studies by Serebrovs’ka [148] confirmed decreased HVR in PD patients without L-DOPA and improvement after the introduction of treatment, which also increased blood DA levels. The results suggested that an impaired DA metabolism in the carotid body affected HVR [135,148]. The possibility that L-DOPA potentiates HVR at the peripheral level supports the concept that DA is an excitatory transmitter in CB [149]. Similar significantly lower chemosensitivity to hypoxia in PD individuals was reported by Onodera et al. [132], who analyzed HVR in patients taking their regular dopaminergic medication (stage II–III) and concluded that the CB dysfunction could be responsible for the coexistence of decreased hypoxic chemosensitivity and blunted perception of dyspnea in PD patients. To exclude factors associated with mechanical ventilation, only patients with normal lung function on spirometry were included in the study [132].

### 7.4. Normoxia, Hypoxia, and Hypercapnia in Animal Studies

Studies on respiratory impairments in patients with PD have many limitations. Patient populations tend to be small and heterogeneous, and long-term follow-up is often not possible to study respiratory dysfunctions from the onset of PD. In addition, patients are usually taking medications, which may interfere with the study results [17]. Therefore, in recent years, animal models of PD, which represent more homogeneous populations and lack dopaminergic supplementation, have been introduced for the study of respiratory disorders. The most commonly used model to induce degeneration of DA neurons in the SNpc and a decline in DA content in the striatum is the injection of 6-hydroxydopamine (6-OHDA) into distinct parts of the nigrostriatal pathway. Unfortunately, the results obtained vary depending on the 6-OHDA application site and the experimental conditions (Table 2).

A decrease in basal frequency of breathing and minute ventilation was observed after bilateral intra-striatal injection of 6-OHDA, which was paralleled by a loss of neurons in the ventral respiratory column including pre-BötC [119]. A unilateral 6-OHDA injection into the MFB with noradrenergic (NA) terminals preservation (desipramine treatment) did not modify the pattern of air breathing [150], suggesting that the unilateral model is unable to provoke abnormalities in normoxic ventilation because the contralateral, intact hemisphere may be sufficient to stabilize the ventilation parameters. An intracerebroventricular (ICV) model with a third brain ventricle 6-OHDA injection caused substantial bilateral DA depletion [117,151] that resulted in decreased basal frequency of breathing and augmented tidal volume [117]. The tidal volume augmentation was explained by a deficit in DA, a neurotransmitter well known to inhibits ventilation during air breathing [152,153]. The most notable change in the baseline respiratory pattern, a reduction in respiratory rate and ventilation, was observed in the reserpine model of parkinsonism [154]. Reserpine is an alkaloid that irreversibly blocks the vesicular monoamine transporter (VMAT), leading to peripheral and central depletion of all monoamine neurotransmitters at synapses [155]. This is somewhat consistent with the bilateral DA deficits obtained in models based on 6-OHDA [117,118], as all studies showed a similarity, in the form of a decrease in basal respiratory rate (Table 2) [117,119,154].

In the bilateral 6-OHDA model, the hypercapnic response is consistent with normoxic changes, namely, a decrease in respiratory rate 40 and 50 days after injury was demonstrated [119]. These results were later confirmed by Oliveira et al. [156], who also noted that the ventilatory response to hypercapnia recovered after 60 days. The altered hypercapnic response was linked to the degeneration of CO_2_-activated periaqueductal gray matter neurons (PAG), which receive projection from the substantia nigra and send projection to the chemosensitive retrotrapezoid nucleus (RTN) [65]. Another study, in which rats were unilaterally injected with 6-OHDA into the MFB, showed increased tidal volume and decreased respiratory rate to a hypercapnic stimulus 14 days after lesion, but minute ventilation remained unchanged [157]. Although a comparison of these studies is virtually impossible because of differences in the models, such as bilateral versus unilateral injections, preadministration of desipramine, and different timing of hypercapnic testing after injury, a similar result is the reduction in respiratory rate stimulation in response to hypercapnia. Similar observations were made in a transgenic model of Parkinson’s disease where Pink1−/− rats presented reduced respiratory rate to hypercapnia (combination of hypoxia) with age [158].

Both 6-OHDA models with unilateral and bilateral DA deficits showed a slight increase in minute ventilation due to a significant increase in tidal volume to the hypoxic stimulus [117,150], which may confirm the inhibitory effect of central DA on the respiratory function. In addition, the lack of effect of peripheral D2 receptor blockade by domperidone in PD rats during HVR may be indicative of impaired CB chemosensitive function in PD [150], as postulated in humans by Serebrovskaya et al., [135]. In contrast to the studies of Andrzejewski et al. [117,150], unchanged HVR was described in rats after 6-OHDA injection into the striatum [119] and in the transgenic PD model consisting of Pink1−/− rats [157]. A significant depression of HVR was demonstrated in the reserpine model [153]. This reduction was likely due to both cerebral and carotid DA deficiency, although in this model, significant muscle stiffness and rigidity may also limit thoracic pump mobility and result in reduced normoxic and hypoxic ventilation [154].

In summary, the ventilatory responses to hypoxia in animal models present varying results, as do studies of idiopathic PD. It all seems to depend on the animal model used and on the experimental conditions. An interesting question arises as to whether the altered ventilatory response to hypoxia, if present in PD, is indeed related to CB dysfunction or to impaired function of the respiratory nuclei and respiratory muscles. Unfortunately, so far, no direct evidence supports any of these concepts.

**Table 2:** Ventilatory responses to hypoxia and hypercapnia in experimental rat models of PD. V_T_; tidal volume, V_E_; minute ventilation, f; respiratory rate.

Model of PD		Normoxia	Hypoxia	Hypercapnia	References
6-OHDA with desipramine pretreatment	unilateral MFB	unhanged	increased V_T_, decreased f, increased V_E_	increased V_T_; decreased f; increased V_E_	[150,157]
6-OHDA with desipramine pretreatment	bilateral ICV	increased V_T_, decreased f,	increased V_T_,	not studied	[117]
6-OHDA	bilateral striatum	decreased f; decreased V_E_	unchanged	decreased f,	[119]
Reserpine with α-ethyl-tyrosine	Intraperitoneal	decreased f; increased V_T_; decreased V_E_	decreased f; increased V_T_; decreased V_E_	not studied	[154]
6-OHDA	bilateral striatum and locus coeruleus	decreased f, decreased V_E_	unchanged	decreased f; decreased V_T_; decreased V_E_	[156]
Transgenic Pink1−/−		increased f	unchanged	decreased f (hypercapnia with hypoxia)	[158]

## 8. Effects of PD Therapy on Respiratory Distress

L-DOPA, the gold standard in PD therapy, is primarily used to treat the motor disorders of PD. However, it can simultaneously affect the respiratory system. The effect of L-DOPA on ventilation is not fully explained, and very often, contentious information appears in the literature. Several studies have found a therapeutic effect of L-DOPA treatment by improving pulmonary functions such as FVC [37,159,160,161], FEV1 [160,161,162], vital capacity (VC) [161], peak expiratory flow (PEF) [159], and respiratory muscle strength [160]. Greater improvement in pulmonary function was noted during the “on” condition in the study of Sathyaprabha et al. [37], while no difference between the “on” and “off” conditions was observed by Tambasco et al. [161]. A detailed summary of articles showing the beneficial effects of dopaminergic therapy on obstructive pattern, restrictive pattern, and upper airway muscles can be found in the reviews by D’Arrigo et al. [56] and Vijayan et al. [26].

Dopaminergic supplementation can affect both function and central coordination of the respiratory muscles. Among other things, dopamine has been shown to have a significant potentiating effect on diaphragmatic contractions [163]. Further, the beneficial effect of long-acting L-DOPA was demonstrated on the apnea rate (reduced apnea–hypopnea index) by administering it to patients overnight compared to the control group [164].

The previously cited study showed that L-DOPA reversed the reduced hypoxic ventilatory response in PD patients [148], which corresponds with an experimental study showing similar results in a reserpine model of parkinsonism [154].

In addition to the beneficial or neutral effects of L-DOPA on ventilation, reports of adverse effects are also present. Among them were described: dyspnea [162,165,166,167], tachypnea [162,166,168], hyperventilation [169], respiratory and diaphragmatic dyskinesias [165,167,170]. Further, brief periods of apnea alternating with irregular tachypnea, consistent with an impaired central respiratory rhythm control, were observed after the peak dose of L-DOPA [168]. In a similar vein, research exists indicating that the administration of dopaminergic supplementation increases the risk of sleep-disordered breathing of central origin [171]. A prolongation of inspiratory duration during L-DOPA therapy in the “on” phase has previously been observed, suggesting that pre-BötC, which plays a key role in the generation of the inspiratory rhythm, may be involved [45].

In addition, respiratory distress may also occur in the “off” state during L-DOPA treatment as a result of rigidity and bradykinesia of the respiratory muscles (Table 3) [172].

Deep brain stimulation (DBS), which alleviates major motor dysfunctions in PD patients, is used when L-DOPA is no longer effective. Unfortunately, a very common side effect is dyspnea associated with the stimulation of adjacent structures, especially present near the bilateral subthalamic nucleus DBS [173,174].

**Table 3:** Effects of L-dopa treatment on breathing.

L-DOPA Positive Effect	L-DOPA Negative Effect
Improvement of FVC, VC, FEV1, and PEF (FEV_1_%, VC%, FVC%) [37,159,161]	Respiratory dyspnea, peak-dose irregular tachypnea alternating with brief periods of apnea, and respiratory dyskinesias [167,168,169]
Increase in respiratory muscle strength parameters [160]	Mild shortness of breath at baseline [175]
Increase in hypoxic ventilatory response [148]	Increased risk of sleep-disordered breathing of central origin [171]
Reduced apnea–hypopnea index (AHI) [164]	In the “off state” of L-DOPA therapy, laryngeal dystonia, stridor, and emergence of chest wall muscle bradykinesia and rigidity [37,61,172,176]

## 9. Conclusions

Respiratory disorders in Parkinson’s disease can manifest as a variety of signs and symptoms. Often unnoticed and neglected by clinicians, they significantly reduce the comfort of patients’ lives; moreover, some of them, such as aspiration pneumonia, may be life-threatening. They have attracted increasing interest from researchers in recent years, who have also examined the effects of movement disorder therapy on breathing. Unfortunately, the study data available to date are either incomplete or contradictory, and respiratory disturbances observed in individuals with PD vary widely, from being asymptomatic to causing sleep-disordered breathing or severe dyspnea. Thus, it seems difficult to draw a clear picture of the respiratory distress present in PD patients as well as to determine its cause. Discrepancies in the depiction of respiratory distress in PD patients may be due to the heterogeneous nature of the disease itself, the comparison of different stages of the disease, the inconsistent inclusion of the effect of antiparkinsonian drugs, and the methodology used. Therefore, further research is needed on the issue of respiratory distress in PD. Studies in animal models could contribute to the elucidation of the pathomechanism of the observed disorders, and although more animal models are emerging, so far a good model that reflects the respiratory disorders observed in humans with PD seems to be lacking.

## Figures and Tables

**Table 1:** Ventilatory responses to hypoxia and hypercapnia in patients with PD.

Treatment	Hoehn & Yahr Scale	Normoxia	Hypoxia	Hypercapnia	References
no data	no data	no data	stimulated	stimulated	[136]
no data	III–IV	unchanged	stimulated	stimulated	[131]
during treatment	I–III	reduced alveolar ventilation	reduced alveolar ventilation	not studied	[135]
during treatment	II–III	no data	reduced	unchanged	[132]
during treatment	I–III	no data	unchanged	reduced	[133]
during treatment	I–II	unchanged	unchanged	unchanged	[134]

## Data Availability

Not applicable.
